# Indirect genetic effects: a key component of the genetic architecture of behaviour

**DOI:** 10.1038/s41598-017-08258-6

**Published:** 2017-08-31

**Authors:** Francesca Santostefano, Alastair J. Wilson, Petri T. Niemelä, Niels J. Dingemanse

**Affiliations:** 1 Research Group Evolutionary Ecology of Variation, Max Planck Institute for Ornithology, 82319 Seewiesen, Germany; 2 Centre for Ecology and Conservation, College of Life and Environmental Sciences, University of Exeter, Cornwall Campus, TR10 9EZ Penryn, UK; 3 Behavioral Ecology, Department of Biology, Ludwig-Maximilians-University of Munich, 82152 Planegg-Martinsried, Germany

## Abstract

Behavioural ecology research increasingly focuses on why genetic behavioural variation can persist despite selection. Evolutionary theory predicts that directional selection leads to evolutionary change while depleting standing genetic variation. Nevertheless, evolutionary stasis may occur for traits involved in social interactions. This requires tight negative genetic correlations between direct genetic effects (DGEs) of an individual’s genes on its own phenotype and the indirect genetic effects (IGEs) it has on conspecifics, as this could diminish the amount of genetic variation available to selection to act upon. We tested this prediction using a pedigreed laboratory population of Mediterranean field crickets (*Gryllus bimaculatus*), in which both exploratory tendency and aggression are heritable. We found that genotypes predisposed to be aggressive (due to DGEs) strongly decreased aggressiveness in opponents (due to IGEs). As a consequence, the variance in total breeding values was reduced to almost zero, implying that IGEs indeed greatly contribute to the occurrence of evolutionary stasis. IGEs were further associated with genetic variation in a non-social behaviour: explorative genotypes elicited most aggression in opponents. These key findings imply that IGEs indeed represent an important overlooked mechanism that can impact evolutionary dynamics of traits under selection.

## Introduction

Behavioural ecologists increasingly focus on studying the adaptive processes maintaining individual differences in behaviour within animal populations. Several adaptive explanations have been proposed for why selection might maintain behavioural variation rather than erode it (reviewed by^1–3^). For example, frequency dependent selection^1^, temporal and spatial heterogeneity^4, 5^, or life-history trade-offs^6–8^ have all been implied to explain the stable coexistence of different behavioural ‘types’ within populations. It is implicitly assumed that genes carried by focal individuals contribute to behavioural differences, such that directional selection should both erode variance and cause a change (over generations) in mean phenotype^9, 10^. However, evolutionary theory also predicts that evolutionary stasis may occur despite directional selection in the presence of ‘indirect genetic effects’ (IGEs) generated by social interactions^11–15^. This key insight has largely been ignored in behavioural ecology theory explaining individual variation in behaviour, despite the fact that many behavioural traits are expressed as part of social interactions.

Quantitative genetic theory implies that social interactions can have major evolutionary repercussions, particularly when an individual’s phenotype is affected by the genotypes of conspecifics: these effects are called IGEs^12, 13, 15^. IGEs can greatly influence evolutionary processes when they are correlated with the direct genetic effects (DGEs) of an individual’s genotype on its own phenotype. For example, in mussel cultures, individuals genetically predisposed to grow quickly in competitive situations are also genetically predisposed to reduce growth in others by depriving them of feeding opportunities^16^. The resulting negative genetic correlation between DGEs and IGEs can impose major evolutionary constraints, by effectively reducing the amount of variation in total breeding value of a trait within a population^17–19^. The presence of IGEs may thus lead to evolutionary stasis in the phenotype, implying that directional selection does not necessarily lead to evolutionary change. Interestingly, positive genetic correlations between DGEs and IGEs are predicted to instead speed up the response to directional selection relative to expectations from classic evolutionary theory (e.g. refs 14 and 15). For example, a positive covariance between DGEs and IGEs on aggression in a study of deer mice (*Peromyscus maniculatus*) implies that this trait  can evolve very rapidly^14^. This is because selection for increased aggression would drive the evolution of a social environment in which aggression is more readily elicited by interacting conspecifics. Therefore, IGEs arising from social interactions can both provide a source of additional genetic variation that either facilitates rapid selection responses or serves as a source of evolutionary constraint on phenotypes^20^. However, to date IGEs have largely been ignored as a potential mechanism explaining evolutionary stasis in individual behaviour research^21–24^.

IGEs are expected to exist on traits such as aggression and dominance^11^, i.e., traits that are expressed explicitly as part of social interactions. Interestingly, IGEs can also affect the evolution of other aspects of phenotype, including behavioural traits not expressed within a social context, provided these covary genetically with traits that do harbour IGEs^25^. For example, the literature on ‘behavioural syndromes’ often reports that traits expressed in social interactions (e.g., aggressiveness, sociability) are phenotypically correlated with other risky behaviours expressed in non-social contexts, such as exploratory tendency, or anti-predator boldness (meta-analysis,^26^). Of course, these correlations are important for evolutionary dynamics only if they are underpinned by genetic processes^10,27^. Thus, if IGEs are present for a social behaviour such as aggression, the evolution of any trait genetically correlated either with the social behaviour or its IGEs may be affected.

Here, we investigated whether IGEs contribute to the genetic architecture of behavioural variation expressed in social and non-social contexts. We repeatedly measured two behavioural traits (exploration, non-social, and aggression, social) in a pedigreed laboratory population of Mediterranean field crickets (*Gryllus bimaculatus*) descended from wild-caught grandparents. For the data presented in this paper, we show elsewhere that exploratory behaviour and aggressiveness are both repeatable and heritable (subject to DGEs) but not genetically correlated (Santostefano *et al*. under review). Here we expand upon these analyses by quantifying (i) whether IGEs also contributed to genetic variance in aggressiveness, (ii) whether, for aggressiveness, DGEs (tendency to act aggressively) and IGEs (tendency to elicit aggressiveness) were correlated, and (iii) whether IGEs on aggression were also correlated with DGEs for exploration, a trait not directly involved in social interactions. Our approach thus implies that drawing evolutionary predictions while ignoring IGEs not only on the focal trait, but also on other seemingly independent traits, can be greatly misleading.

## Results

### Sources of variation in single traits

Exploration behaviour was significantly repeatable (*r* = 0.45) and heritable (h^2^ = 0.28) (see also Santostefano *et al*. under review). Aggressiveness was also significantly repeatable (*r*_*f*_ = 0.17) and heritable (h^2^ = 0.05), while it additionally harboured a significant opponent identity effect (*r*_*o*_ = 0.11) (see also Santostefano *et al*. under review; estimates re-printed in Table 1). Here we expanded upon these analyses by estimating IGEs on aggression and testing for their correlation with DGEs. Doing so, demonstrated that this opponent effect harboured a small, but significant, amount of genetic variation for focal aggression (V_IGE_ = 0.026, SE 0.017) (Model 6, Table 1). In other words, there was genetic variation not just in the tendency of individuals to be aggressive, but also in the level of aggressiveness they elicited in their social partners. Furthermore, the genetic correlation between DGEs and IGEs for aggression was strong and negative (r_G_ = −0.83, SE 0.37) (Model 7, Table 1). AIC model comparison to simpler models also provided strongest support for this final model (Model 7, Table S1). In other words, individuals genetically predisposed towards expressing higher levels of aggression as a focal were also predisposed to suppress aggressiveness in their opponents. As a consequence of this tight negative genetic correlation, the estimated total heritable variation in aggression (also known in the literature as τ^2^ ^28^) (V_TBV_/V_TOT_ = 0.016, SE 0.030; where V_TBV_ = V_DGE_ + V_IGE_ + 2COV_DGE,IGE_ = 0.051 + 0.026 − 2*0.030 = 0.016; V_TOT_ = 0.99) was considerably smaller (namely, 3.19 times) than what ‘traditional’ estimates of heritability based on DGEs would (inappropriately) conclude (h^2^

**Table 1:** Results of the univariate mixed ‘animal model’ fitted to partition variation in aggressive behaviour with random intercepts for focal and opponent identity. Estimates of variance components and their correlations are given with associated standard errors. Random effects are expressed as the proportion of total phenotypic variation not attributable to fixed effects explained by each effect. Focal and opponent variances, as well as their covariance, are partitioned into environmental (PE) and genetic (G) components. For each model, variance terms are provided with a likelihood ratio test (LRT) between the given model and the previous model, with associated degrees of freedom (df) and values of P. The most parsimonious model (model 7) is denoted in bold face.

**Model**	**Variance σ² (SE)**							**Correlations r Foc-Opp (SE)**			**Test**			
	**Focal**			**Opponent**			**Residual**		**PE**	**G**	**LogL**	**Χ²**	**df**	**P**
		**PE**_**(f)**_	**DGE**		**PE**_**(o)**_	**IGE**								
1	—	—	—	—	—	—	0.98 (0.03)	—	—	—	−1168.01	—	—	—
2	0.17 (0.02)	—	—	—	—	—	0.83 (0.02)	—	—	—	−1131.75	72.52	0/1	<0.01
3	0.17 (0.02)	—	—	0.11 (0.02)	—	—	0.71 (0.03)	—	—	—	−1116.89	29.27	0/1	<0.01
4	0.17 (0.02)	—	—	0.11 (0.02)	—	—	0.71 (0.03)	−0.21 (0.11)	—	—	−1115.34	3.1	1	0.08
5	—	0.12 (0.03)	0.05 (0.02)	0.11 (0.02)	—	—	0.71 (0.03)	−0.19 (0.14)	—	—	−1110.99	8.7	0/1	<0.05
6	—	0.12 (0.03)	0.05 (0.02)	—	0.08 (0.03)	0.03 (0.02)	0.71 (0.03)	−0.20 (0.15)	—	—	−1109.15	3.68	0/1	<0.05
**7**	—	**0.12** (0.03)	**0.05** (0.02)	—	**0.08** (0.03)	**0.03** (0.02)	0.71 (0.03)	—	0.01 (0.18)	**−0.83** (0.37)	−1107.05	4.2	1*	<0.05

^*^tested in addition over an equal mix of df = 1 and df = 2 (representing a test of variance and covariance together, against model 5), Χ² = 7.88, p < 0.05

= 0.051, SE 0.024).

### Among-trait correlations

Multivariate models corroborated the strong negative genetic correlation between DGEs and IGEs on aggression (r_G_ = −1.02, SE 0.40, P < 0.05) (Table 2). We note this estimate is slightly greater than that presented above (though based on SE the confidence intervals will be strongly overlapping) and very slightly outside the permissible parameter space for a (true) correlation (we also note that not constraining the parameter space in the model fit allows better convergence and an estimate of the uncertainty associated with r_G_). However, the genetic correlation between DGEs on exploration and DGEs on aggression was close to zero and non-significant (r_G_ = −0.04, SE 0.24, P > 0.05) (Table 2), contrary to predictions from the behavioural syndrome literature. Multivariate models also provided some evidence for a positive genetic correlation between IGEs on aggression and DGEs expressed in the non-social trait of exploration, although the estimated was marginally non-significant (r_G_ = 0.59, SE 0.28, P = 0.056) (Table 2).

**Table 2:** Estimated additive genetic (**G**) covariances and correlations (with SE) between two behaviours (aggression and exploration), and IGEs on aggression. We present covariances (lower-off diagonals) and correlations (upper-off diagonals) for each set of traits. Correlations printed in bold-face are significant (P < 0.05) based on likelihood ratio tests derived from the multivariate model detailed in the main text.

**G**	**Aggressiveness (DGE)**	**Exploration (DGE)**	**Aggressiveness elicited (IGE)**
Aggressiveness (DGE)	—	−0.04 (0.24)	**−1.02** (0.40)
Exploration (DGE)	−0.01 (0.03)	—	0.59 (0.28)
Aggressiveness elicited (IGE)	−0.04 (0.02)	0.05 (0.03)	—

**Figure 1:**
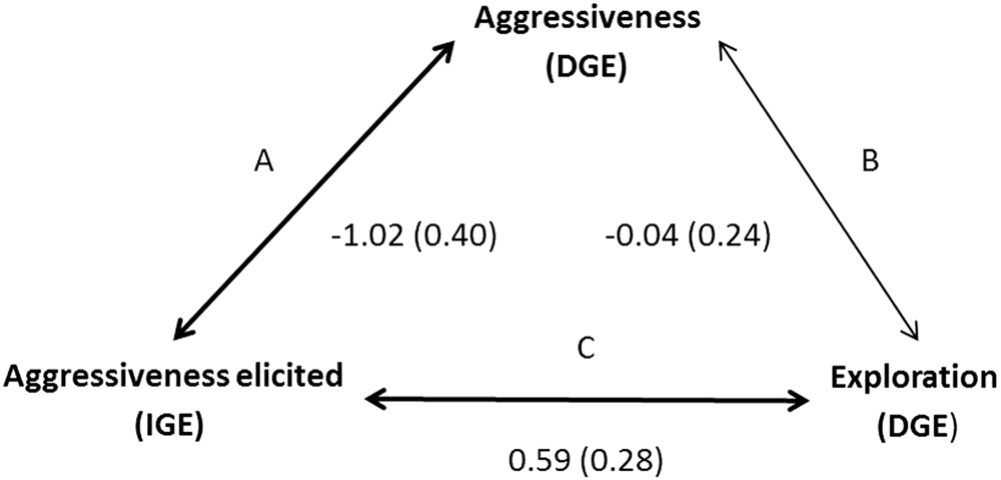
Correlation structure of the five hypothesized multivariate model structures presented in Table 3 (detailed in the Methods). A is the correlation between DGEs and IGEs on aggressiveness; B is the correlation between DGEs on exploration and DGEs on aggressiveness; C is the correlation between DGEs on exploration and IGEs on aggressiveness. Estimated correlations with corresponding SEs derived from the full model (Model 5, presented in Table 2) are shown with each arrow; bolded arrows represent paths with statistical support from the LRT and AIC.

Using the estimated **G** matrix, we compared the fit of five model structures (considered a priori) using AIC (Table 3, Fig. 1). This approach is warranted because a multivariate rather than a pair-wise bivariate approach greatly increases statistical power. A model where both the correlation between DGEs on exploration and IGEs on aggression, as well as the correlation between DGEs and IGEs on aggression were included (Model 3) fitted the data best, consistent with our inferences from likelihood-based testing of the pairwise correlations (above) (Table 3, Fig. 1). The direct genetic correlation between aggression and exploration was not included in this model, consistent with this correlation being close to zero in the full model estimated above. This full pattern is somewhat difficult to interpret since, given the magnitude of estimated correlations between IGEs for aggression and DGEs on both behaviours, we might have expected a stronger (direct) genetic correlation between aggression and exploration. As this was not the case, it is possible that the IGEs and DGEs for aggression are not as tightly correlated as implied by the point estimate (see also our discussion above). With this caveat noted, we find by AIC comparison that individuals with a high genetic merit for explorative tendency in novel environments tended to elicit more aggression (Table 3, Fig. 1). Taken together with the strong (and significant) genetic correlation between DGEs and IGEs on aggression (Table 1; Table 2), we view this as evidence that the social environment can indeed influence the evolution of behaviours including those expressed outside the social context.

**Table 3:** Relative fit of five multivariate models differing in architecture of genetic correlations between direct genetic (DGE) and indirect genetic (IGE) effects based on the Akaike’s information criterion (AIC). We present each model’s AIC-value relative to the model with the lowest AIC-value (ΔAIC), its weight, and relative likelihood. Model denominations refer to Fig. 1: A is the correlation between DGEs and IGEs on aggressiveness; B is the correlation between DGEs on exploration and DGEs on aggressiveness; C is the correlation between DGEs on exploration and IGEs on aggressiveness. Model 5 (the complete model) is presented in Table 2.

**Model**	**ΔAIC**	**Akaike Weight**	**Relative LL**
3. B = 0	0	0.78	1.00
4. C = 0	3.62	0.13	0.16
5. A, B, C estimated	5.49	0.05	0.06
1. A, B, C = 0	6.06	0.04	0.05
2. A = 0	8.64	0.01	0.01

## Discussion

This study investigated a largely overlooked mechanism, indirect genetic effects, which may contribute to the observed behavioural variation in social traits under selection and impact their evolutionary dynamics. Our study on male Mediterranean field crickets confirmed that the phenotypic expression of aggression and exploration was repeatable, and showed that the former depended on opponent, as well as focal identity. Both behaviours harboured additive genetic variance, but—importantly—heritable variation in focal aggressiveness arose jointly from the genotypes of the focals (DGEs) and opponents (IGEs) (Table 1). As aggressiveness represents an important component of an often-documented “aggression-boldness syndrome”^26^, the evolutionary consequences of these IGEs may extend to other associated traits. Indeed, we found evidence for a genetic architecture suggesting that the evolution of a non-social trait such as exploration may not be independent from the evolution of a social trait, and vice versa, given that its DGEs were correlated with the IGEs acting on aggression. Our study therefore identifies IGEs as an important overlooked component of the (multivariate) genetic architecture of behaviour that should be considered when making predictions on the evolution of traits studied in ‘personality’ research. Our results generally imply that IGEs can have consequences for the evolutionary trajectories of a wide range of traits, including those not expressed as part of social interactions (e.g., exploratory tendency, body size, etc.).

The estimated magnitude of IGEs on aggression in this study was similar to that documented in other species (e.g. refs 14 and 29). Crucially, we also found a strong negative correlation between DGEs and IGEs for this interactive behaviour, a result that contrasts with positive correlations reported for agonistic behaviours in some other species^14^ (but not all^29^). An important consequence of the strong negative covariance between direct and indirect genetic effects is that the total heritable variation for aggressiveness is reduced^17, 18^. This is highlighted in our results by the discrepancies between the (direct) heritability estimates (h^2^ aggression = 0.051), and the total heritable variation for aggression including IGEs and their covariance with DGEs (τ^2^ = V_TBV_/V_TOT,_ = 0.016). While indirect effects (genetic and non-genetic component) clearly contribute to variance in focal aggressiveness, the negative correlation between IGEs and DGEs means that the potential for evolution of the phenotypic mean in response to directional selection is even lower than suggested by the (direct) heritability^20, 30^.

The sign of this correlation can also be interpreted in terms of behavioural feedback processes and the functional role of aggression. For example, in species (or contexts) where individuals escalate agonistic behaviour through positive feedbacks (i.e. aggression elicits aggression^14^) direct-indirect (genetic) covariance will be positive. Conversely, negative correlations arise when aggression is asymmetric, being directed by more competitive (or dominant) individuals towards subordinate social partners. This is because, in a dyadic contest, a genotype predisposing to contest winning by the focal will necessarily predispose to losing when encountered in an opponent^20,28,31, 32^. Thus, the negative genetic correlation found here actually suggests that, at least within the context of the behavioural trials conducted, aggression is being used to assert social dominance in this species. The importance of such correlations applies to any species displaying aggressive interactions, regardless of whether aggression is part of stereotyped escalated context or linked to dominance.

A question not previously considered is whether IGE on aggression (or indeed other social traits) will also have evolutionary implications for non-social aspects of ‘animal personality’^23^. For example, traits such as boldness and exploratory tendency are often correlated with aggression (e.g. mediated by proximate mechanisms such as variation in metabolism^7, 8^), leading to the suggestion of an integrated ‘aggression-boldness syndrome’ (meta-analysis, ref. 26). When we thus extended our analysis to include a non-social behaviour, we found evidence of a genetic covariance structure that would preclude independent evolution of exploration and aggressiveness. Interestingly, this was manifest as a correlation between IGEs on aggression and DGEs for exploration, rather than the conventional (i.e. direct additive) genetic covariance structure that is normally estimated in studies seeking to understand multivariate selection responses (e.g. using the Lande equation, refs 15 and 33). Specifically, a high genetic merit for exploration is associated with a tendency to elicit more aggressive behaviour from conspecific partners (Table 2, Fig. 1). The correlation between DGEs in exploration and IGEs in aggression mirrors, at the genetic level, conclusions of a phenotypic study on the closely related cricket species *G. campestris* ^24^. In this species we found a positive correlation between individual (phenotypic) merits for exploration and aggression elicited in conspecifics (r_I_ = 0.45, SE 0.17) (Note the corresponding among-individual phenotypic correlation estimated in the present experiment is also significantly positive and similar in magnitude: r_I_ = 0.37, SE 0.09; Table S2). Thus, had we not considered IGEs, we would incorrectly have concluded that exploratory behaviour and aggressiveness were evolutionarily independent^10, 34^. Instead we expect that selection on exploratory behaviour will cause correlated evolution of the social environment with consequences for mean aggression (and vice versa). However, it does not follow that the IGEs constraining evolution of mean aggression will necessarily constrain the evolution of exploration behaviour too. In general, IGEs arising from competition related processes are expected to impose constraints on traits that are consequent, rather than causal to, contest outcomes (and thus resource acquisition^35^), a scenario that is not clearly the case here. We fully acknowledge that our study is not directly informative for the causal pathways linking aggression to exploration, but several possibilities can be hypothesised. For example, the positive association could arise if exploration in a novel environment increases the likelihood of encountering rivals (and thereby provoking more attacks from conspecifics). Exploration could also be favoured in individuals eliciting aggression as a result of competition for territories in the population. Alternatively, exploratory tendency may be (genetically) correlated with other traits that directly mediate agonistic behaviour in competitive interactions (e.g. size, weapon morphology).

We also note that the variance partitioning approach used to model IGEs in this paper is mathematically equivalent to the alternative (but complementary) ‘trait based’ approach advocated by others^15, 18, 36^. In this latter framework, an interaction effect coefficient ψ (‘psi’), captures the effect of a measured conspecific trait (or traits) on focal phenotype. ψ represents a standardized reaction norm slope, hence the level of phenotypic plasticity to a social environmental gradient^11^. In the context of our study, ψ is captured by the correlation between DGEs and IGEs: individuals responded to the aggressiveness and explorative tendency expressed by social partners (because IGEs on aggression are correlated to DGEs of both behaviours), implying that ψ is multivariate in nature. A hot question in quantitative genetics revolves around the issue of whether genotypes differ in their responsiveness to phenotypes of conspecifics, which would imply heritable variation in ψ^37, 38^. An interesting follow-up question is thus whether responsiveness to other individuals (ψ) varies according to behavioural ‘types’, as has recently been suggested in the personality literature^23, 39^. Importantly, a genetic architecture that includes genetic variation in ψ and its covariance with other DGEs and IGEs would likely reveal further interesting repercussions for evolutionary processes of behavioural traits.

In conclusion, a crucial consequence of social interactions is that they generate IGEs that not only contribute to the observed variance but also impact evolutionary dynamics of traits under selection. In this case, constraints on the phenotypic evolution of mean aggression arise from the negative correlation of direct and indirect genetic effects. More generally, we note that the role of IGEs has received little attention in ‘animal personality’ research, despite their potential implications for generating (and possibly maintaining) among-individual behavioural differences. The merit of our approach is that by including IGEs into behavioural ecology’s existing ecological frameworks to study ‘personality’, we may finally start fully integrating distinct areas of evolutionary biology such as quantitative genetics and behavioural ecology^23, 40^. Doing so allows us to address outstanding questions about the evolution of behaviour. Importantly, this heuristic framework may be broadly applied to any trait associated with traits involved in social interactions. Indeed, traits such as coloration, ornaments, badge of status, are often correlated with aggression or dominance^30^. More generally, our study also demonstrates the importance of viewing the phenotype (or genotype) from a multivariate perspective. That is, predictions of how ‘personality’ traits respond to selection can be profoundly misleading if effects of social interactions mediated by IGEs are not considered.

## Methods

### Cricket collection, breeding, and housing

The parental generation of crickets was collected from a tomato field of approximately 2500 m^2^ near Capalbio, Italy (42°42′46.7′ N 11°33′99.3′ E) in July 2013. We collected a total of 100 individuals: 34 adult males, 33 adult females, 12 near-final instar males, and 21 near-final instar females. Following capture, crickets were transported to a climate controlled chamber at the Ludwig Maximilians University of Munich (Planegg-Martinsried, Germany), where they were housed at 26 °C ( ± 0.5) and 65% ( ± 0.5) humidity, under a 14:10 light:dark photoperiod (h) that wild crickets experienced at the time of capture.

Sexually mature wild-caught individuals from the parental generation were randomly paired 4 days after arrival in the laboratory. A total of 35 males and 35 females produced a total of 34 clutches from which offspring hatched. We raised 40 offspring (F1) per parental pair (1360 offspring in total), from which we randomly selected breeders once reaching adulthood. We adopted a full-sib/half-sib breeding design^41^ for the F1 and F2 generations by having each male fertilize the clutches of two females. We used a total of 35 males and 70 females from the F1 generation, and 15 males and 30 females from the F2 generation. This resulted in 47 F2 and 21 F3 viable full-sib families. Details on the breeding and rearing protocol are provided in the Supplementary Material.

Adult males of the F2 and F3 generation were subjected to repeated behavioural assays. The study focused on males only because aggression through escalated stereotyped fights is largely male-limited, thus more difficult to measure in females. The number of available adult offspring (of both sexes) per female was n = 622 for the F2 and n = 281 for the F3 (per female mean ± SD: 8.64 ± 2.46 for the F2 and 5.51 ± 2.44 for the F3). Of these, a total of 455 males were selected and screened for behavioural phenotypes (335 from the F2 and 120 from the F3).

### Experimental protocol

Behavioural trials were conducted between January and June 2014. Each individual was repeatedly assayed for each of 2 behaviours on the same day (exploration and aggression, described in detail below) following^24^; the same individual was assayed for each behaviour 6 times, with measurements taken approximately one week apart (range 7–9 days). Because individual identification is required for the aggression test (detailed below), subjects were marked with coloured tape on the pronotum (red or blue, randomly assigned each time) the day before a focal trial (see also^24^). The two tests were always done sequentially and in the same order; carry-over effects could therefore not be modelled. We chose this set-up because it ensured that all individuals were given the exact same treatment since this greatly facilitates comparison between individuals^42, 43^.

The 455 males were divided into 7 groups of 40 individuals (F2), one group of 55 individuals (F2), and 3 groups of 40 individuals (F3). 15 individuals of the F2 were only tested twice, because they were subsequently used for other purposes. Individuals were divided into groups according to their estimated age (days post-moulting) to avoid any possible age-related effects on aggression (see also^24^). All individuals within a group were tested on the same day (8 individuals simultaneously), randomized for time of the day and test location. Dyads of males paired for the aggression tests were randomly assigned amongst the non-related individuals within the same group to produce social environments that were homogenous with respect to relatedness.

All trials were performed on a rack fitted with two shelves, each equipped with a camera, in the same climate room where the individuals were housed (detailed in ref. 24. All trials were recorded using high-resolution digital video cameras (Basler GenICam, Germany) fitted 43 cm above each testing arena. The cameras were connected to a computer outside of the climate room and managed using the software MediaRecorder (Noldus, Netherlands). Videos were recorded at 27.81 frames per second and 1600 × 1200 pixels resolution.

A small number of trials were excluded from the final dataset: 31 of 1888 (F2) and 3 of 608 (F3) for exploration trials (respectively 1.64% and 0.49%), and 27 of 944 (F2) and 5 of 304 (F3) for aggression trials (respectively 2.86% and 1.64%) due to technical problems with data recording or video-tracking. Note that the total number of aggression trials is approximately half of that of other trials since two individuals are involved in each aggression test. The final sample size (behavioural tests) was therefore 2462 for exploration (mean number per individual: 5.27, SD 1.23) and 1195 for aggression (mean number per individual: 5.16, SD 1.28) tests.

### Behavioural trials and scoring

Exploration and aggression behaviour were assayed following the protocol in^24^ (for an illustration of the setup, see Fig. 2 in^24^). Briefly, at the onset of the exploration test, each individual was moved (inside its own shelter) from its home container to the exploration arena. Exploration activity was then recorded automatically for 30 minutes. Following the exploration test, the shelters were removed and the individuals given a further 10 minutes to acclimatize. The divider between two arenas was then lifted, after which we filmed each dyad engaging in social interactions for a period of 10 minutes. We then returned the crickets to their home containers in the allotted housing slots within the climate room.

Exploration and aggression videos were analysed using Ethovision version 11.0 (Noldus, the Netherlands). This software package enables tracking of isolated individuals and extracts the spatial coordinates for each video frame. We summed up all distances to calculate the total distance moved in the novel environment (exploration test), viewed as proxy for ‘exploration behaviour’ (following^44^). For the aggression test, we calculated the total time each individual spent moving towards the opponent (‘relative movement’ for simplicity), by summing up only the consecutive samples (frames) where the relative distance between subjects decreased (see User manual of Ethovision v11.0, Noldus Information Technology 2014, for details). We set a maximum interaction distance between the two subjects of 8 cm based on pilot trials to define a range in which the directional movement towards the other cricket would be meaningful. We validated the choice of the variable ‘relative movement’ for aggression both for a related cricket species and for a subset of the current dataset. Relative movement was highly correlated with the variable ‘approach’ towards the opponent that we scored manually, and is commonly used in aggression tests^44^. The choice and validation of relative movement as a measure for aggression is detailed in the Supplementary Material.

### Quantitative genetics analysis

#### Univariate models

We conducted two sets of statistical analyses. First, using univariate mixed-effects models we partitioned the total phenotypic variance (V_P_) for each measured trait (aggression, exploration) into its underlying components: residual within-individual variance (V_R_) and among-individual variance (V_I(f)_) for the focal individual. The latter component represents the statistical signature of “personality” variation^45^ and so was tested in its own right before we further partitioned it in another model into direct (additive) genetic (V_DGE_) and permanent environmental (V_PE(f)_) effects. For aggression, we also estimated the variance explained by the opponent identity (V_I(o)_), which was, in turn, also split into its environmental (V_PE(o)_) and genetic (V_IGE_) components. Partitioning of genetic from non-genetic focal (direct) and, for aggressiveness, opponent (indirect) variances was done using a univariate mixed-effects “animal” model^46^ that utilised the (additive) relatedness matrix determined from the pedigree. Covariance between direct and indirect effects was modelled in both genetic and permanent environment parts of the model. Behavioural data was available for both partners in every dyadic aggression trial, meaning the designations of focal and opponent within a dyad are arbitrary. Thus, for a two individuals in a dyad (i, j), we model the indirect effect of j on i’s phenotype and vice versa (i.e. each dyad contributes two focal records). We note that a possible issue arises since residuals are likely to be correlated between the two observations per dyad, but since the correlation is likely negative where aggression reflects dominance, this is not readily accounted for by modelling a random effect of dyad. We therefore blocked the data file into two “realizations” of focal versus opponent designation, each block containing focal records on one individual within each dyad. The two data blocks were then analysed simultaneously within a single mixed model formulation, with no cross-block covariance terms fitted, but under an imposed constraint that within-block (co)variance components to be estimated are equal in the two data blocks. More detail and ASReml code to implement this modelling strategy is provided in the Supplementary material.

To statistically control for sources of variation in behaviours not directly relevant to our hypotheses, we included the following fixed effects: test sequence (covariate, range 1–6, mean centered), generation (F2 or F3) and clutch number (first or second) (both coded as −0.5 and 0.5, following^47^). All models were fitted using restricted maximum likelihood; dependent variables were mean-centred and variance standardized to facilitate comparison of variance components across traits. Throughout, we assumed a Gaussian error distribution, which was confirmed for all response variables after visual inspection of model residuals.

Adjusted individual repeatability^48^ was estimated for each behavioural trait by calculating the proportion of the total phenotypic variance not attributable to fixed effects that was explained by among-individual variance (i.e., where V_I(f)_ = V_PE(f)_ + V_DGE_). For aggression, we estimated both focal and opponent repeatabilities. Direct heritability (h^2^), indirect genetic effects (IGEs), and the proportional contribution of V_PE(f)_ (pe_(f)_^2^) and V_PE(o)_ (pe_(o)_^2^) relative to the total phenotypic variance were estimated as each variance component divided by total phenotypic variance not attributable to fixed effects. From this latter model, we further calculated the variance in total breeding value (V_TBV_) for aggression. V_TBV_ allows estimating the total heritable variation for this trait available to selection, taking into account DGEs, IGEs, and their genetic covariance. V_TBV_ was calculated following^28^ (eq. 6, for a group size of two interacting individuals, n = 2) as V_TBV_ = V_DGE_ + V_IGE_ + 2COV_DGE,IGE_. We calculated the total heritable variation for aggression as τ^2^ = V_TBV_/V_TOT_^28^.

#### Multivariate models

As a next step, we used a multivariate extension of the framework described above to estimate patterns of between-trait (aggression, exploration) covariance at the among-individual (**I**) level, further partitioned into the permanent environmental and genetic levels by respectively estimating the **PE** and **G** matrices. This allowed us to estimate the correlation between the opponent identity effect on aggressiveness and the focal identity effect on exploration (**I** matrix), and to partition it into its genetic and environmental components. We fitted exploration and aggression as response variables and included only fixed effects that explained significant variation in univariate analyses (detailed above).

#### Significance testing in mixed-effects models

We tested model fixed effects using conditional F-tests with denominator degrees of freedom (df) estimated from the algebraic algorithm in ASReml 4.1^49^. We used a hierarchical stepwise forward approach^50, 51^ to evaluate the statistical significance of random effects by likelihood ratio tests (LRTs). We started with a phenotypic model that contained only fixed effects and residual variation (Model 1). We then tested for differences among individuals in the focals (Model 2) and the opponents (Model 3) by sequentially fitting individual and opponent identities respectively. Model 4 tested for the phenotypic correlation between the two. We repeated the same structure when testing for genetic variation and added DGEs (Model 5), IGEs (model 6), and their correlation (model 7). We assumed a χ^2^-distribution for the test statistic which is calculated as twice the difference in log-likelihood between a model where a target random effect was fitted versus not fitted^52^. Variances are bound to be positive, therefore in testing them we applied the LRT assuming (for testing a single variance component) an equal mixture of χ^2^_0_ and χ^2^_1_^53–55^.

For multivariate models, we compared the fit of a model where all covariances at a specific level were estimated with one where those covariances were instead all constrained to zero (with degrees of freedom equal to the number of covariance terms). This provides an overall (i.e. matrix level) test for nonzero covariance structure. We further tested the significance of each covariance separately by applying a LRT (assuming χ^2^_1_) as described above. This led to 5 alternative multivariate models, differing in the correlation structure (See Table 3 for details). We also compared the fit of the alternative models (both for univariate and multivariate analyses separately) using the Akaike information criterion (AIC)^56, 57^, calculating ΔAIC relative to the model with the lowest AIC. We calculated the Akaike weight and model likelihood for each model^58^ using the package ‘qpcR’^59^ in R 3.1.0^60^.

## Electronic supplementary material

Supplementary Information
