# Supplementary material for: Indirect genetic effects: a key component of the genetic architecture of behaviour
Source: Sci Rep. 2017 Aug 31;7:10235. doi: 10.1038/s41598-017-08258-6 (PMC5578976; doi:10.1038/s41598-017-08258-6)
Supplement: Supplementary file 1 — Supplementary Information [file 41598_2017_8258_MOESM1_ESM.doc]

**SUPPLEMENTARY INFORMATION**

Title:

Indirect genetic effects: a key component of the genetic architecture of behaviour

Authors:

Francesca Santostefano¹, Alastair J. Wilson², Petri T. Niemelä^3^, and Niels J. Dingemanse¹,³*

**SUPPLEMENTARY TEXT**

**Breeding and rearing protocol**

Each adult male (‘sire’) was mated twice with each of two unrelated females (‘dams’) to ensure offspring production with each female in case the first clutch failed. Mating took place inside a plastic box (10×8×14 cm^3^) equipped with a cardboard shelter, *ad libitum* food and water, and a plastic cup (diameter × height: 7×4.5 cm^2^) filled with moist humus for oviposition. The male was moved after 3 days to the mating box of the second female; at the same time, the oviposition cup of the first female was moved to a plastic box (6×9×9 cm^3^), where the eggs hatched on average after 13.04 (SD 2.63) days. Provided that ≥50 offspring hatched from the first clutch, we discarded the second egg batch. If not, we used offspring from the second egg batch for our experiments. 5-6 days following hatching, we counted the nymphs in each box and placed 20 randomly chosen offspring in each of two new plastic rearing boxes (13×15×22 cm^3^). In other words, 40 offspring per full-sib family were taken forward. Each rearing box contained a carton shelter, water and food *ad libitum,* and a substrate of fine pebbles and sand. After 5 weeks, containers were checked daily for final instars nymphs, which were subsequently removed and housed individually awaiting sexual maturation. Adult individuals were housed alone in a plastic container (10×10×9 cm^3^) with a sand-covered floor and a flow-through plastic netted lid that prevented escape but allowed air circulation. Each container included an artificial, half-cylindrical shelter (6×3.5×2 cm^3^), a petri dish (with a diameter of 3.5 cm) with food, and another petri dish with water held within a cotton-plugged vial. Individuals were fed with a mix of dry bird food (Aleckwa Delikat, Germany) and fresh slices of apples *ad libitum*. Food and water were replaced every 3-4 days. Individuals were kept in these same conditions until natural death (F2 generation) or until they were euthanized at the end of the experiment by placing them in a -20°C freezer (F3 generation).

**Validation of aggression measurements**

The choice of relative movement as a measure for aggression was taken in two steps. First, we explored, for a published dataset obtained from a related species (*G. campestris*) (Santostefano et al. 2016), how various candidate metrics (automatically derived from our tracking software) predicted aggression, which we defined, and scored manually, as ‘approach’ towards the opponent. We scored an individual as ‘approaching’ during an interaction when it moved towards the other individual from any angle until they came into contact. When only one individual was actively approaching the other (i.e. the other cricket sat still), we assigned the behaviour to that individual alone. In cases where both contestants approached each other at the same time, we assigned the behaviour to both. Amongst the automatically-derived candidate metrics, ‘relative movement’ provided the highest correlation with this manually scored measure of aggression (r = 0.85, 0.03 SE). We therefore selected this metric and validated its correlation with aggression (i.e., approach) in a randomly chosen subsample of the *G.bimaculatus* dataset presented in the current paper, where the correlation was indeed satisfactory (r= 0.80, 0.06 SE, n = 30 videos). This independent confirmation therefore supported the notion that ‘relative movement’ represented a reliable measure of aggression, and we used this automatically-tracked measure of aggression for the data analyses presented in the current paper.

**ASREML annotated code**

As detailed in the main text, analyses of aggressiveness that estimate focal and opponent identity effects typically focus on variation in the behavior expressed by the (arbitrarily assigned) focal individual alone. Here we detail how we incorporated information on the same behavior measured on the opponent in the statistical model while avoiding pseudo-replication. We started with the following data structure, where each line consisted of information regarding the identity of both individuals, one arbitrarily called ‘Individual A’ and the other ‘individual B’, with associated information regarding their aggressiveness:

| **trial ID** | **Individual A** | **Individual B** | **Aggressiveness A** | **Aggressiveness B** |
| --- | --- | --- | --- | --- |
| 1 | 14 | 12 | 3 | 6 |

We then rearranged the data in the following way:

| **trial ID** | **Focal** | **Opponent** | **Data block** | **Aggressiveness 1** | **Aggressiveness 2** |
| --- | --- | --- | --- | --- | --- |
| 1 | 14 | 12 | 1 | 3 | NA |
| 1 | 12 | 14 | 2 | NA | 6 |

In this re-ordered dataset, the data is printed over two lines, once viewing individual A as the ‘focal’ individual in trial 1 (assigned to Data block 1) and once viewing individual B as the ‘focal’ individual in trial 1 (data block 2). Importantly, the behavior of the individual dubbed ‘focal’ in Data block 1 was printed in another column (Aggressiveness 1) than the behavior of the individual dubbed ‘focal’ in Data block 2 (column Aggressiveness 2). Analysis of either trait (Aggressiveness 1 or 2) alone would yield valid estimates of model parameters relating to DGE and IGE for aggressiveness. However, the estimate would not be informed by all available data. We therefore formulated a bivariate analysis under the imposed condition that all parameter estimates (fixed effect coefficients and (co)variance components) are equal for the two homologous traits as defined in the two data blocks (i.e., Aggressiveness 1, Aggressiveness 2). Practically this can be achieved for a pair of homologous traits by fitting a bivariate mixed effect model with the following code in ASReml, which we have annotated in footnotes below. Note for simplicity the code below has only a mean in the fixed effects part of the model.

agg1 agg2~mu !r !{Trait.foc Trait.opp !} !{Trait.ide(foc) Trait.ide(opp) !}

1 2 2

0

Trait 0 US !GPZP !=a0a !S2==1 #A

0.5

0 0.5

Trait.foc 2

4 0 US !GPZPUZPZUZP !=a0ab0c0b0c #B

0.5

0 0.5

0.1 0 0.5

0 0.1 0 0.5

foc

Trait.ide(foc) 2

4 0 US !GPZPUZPZUZP !=d0de0f0e0f #C

0.5

0 0.5

0.1 0 0.5

0 0.1 0 0.5

ide(foc)

Footnotes:

The !{Trait.foc Trait.opp !} command enables joining the focal and opponent variance-covariance matrix into a single matrix such that covariances between focal and opponent identity effects can be estimated.

A – Residual covariance structure (R). Residual variances are constrained to be positive and equal for the two traits. A starting value of 0.5 is supplied. Since no line of data is informative for both traits the residual covariance is not estimable and is fixed (arbitrarily) to zero.

B – Genetic covariance structure (G). There are four random effects in the model (focal and opponent effects on two homologous traits) so a 4x4 covariance matrix is specified. Variances are constrained to be positive (starting value of 0.5 supplied for each), while covariance terms are identifiable between focal and opponent effects with each trait (starting value of 0.1 supplied). Covariance parameters for Aggression 1 are constrained to equal those of Aggression 2. All cross-block covariance terms are fixed to zero.

C –Permanent environmental covariance structure (PE). There are four random effects in the model (focal and opponent effects on two homologous traits) so a 4x4 covariance matrix is specified. Variances are constrained to be positive (starting value of 0.5 supplied for each), while covariance terms are identifiable between focal and opponent effects with each trait (starting value of 0.1 supplied). Covariance parameters for Aggression 1 are constrained to equal those of Aggression 2. All cross-block covariance terms are fixed to zero.

*Alternative approaches:* The modeling procedure detailed above has the main advantage of allowing the information on the behavioural phenotypes of both contestants to be used in statistical analyses. We also considered a simpler alternative approach that would seemingly achieve the same aim. In this approach, the behavioural phenotypes measured for both contestants would be fitted as separate data points (lines) but placed within a single column:

| **trial ID** | **Focal** | **Opponent** | **Data block** | **Aggressiveness** |
| --- | --- | --- | --- | --- |
| 1 | 14 | 12 | 1 | 3 |
| 1 | 12 | 14 | 2 | 6 |

Importantly, with this arrangement, appropriate statistical analyses should consider the possibility of residual covariance between the focal and opponent behavior. A positive residual covariance would, in this arrangement, lead to a trial identity effect when fitted as a random effect. By contrast, a negative residual covariance cannot be modelled with this arrangement, which is problematic as it is likely to exist (i.e., trials where one individual is relatively aggressive, the other is relatively less aggressive). Importantly, as detailed above, our approach does not require fitting this residual covariance as it is non-identifiable because of the way that the data is arranged; this alleviates this important concern. We thus view our approach is heuristic as it does not require additional assumptions to be made.

**SUPPLEMENTARY TABLES**

**Table S1.** Relative fit based on the Akaike’s information criterion (AIC) of the seven univariate mixed models presented in Table 1. These models partition variation in aggressive behaviour and differ in random effects structure. We present each model’s AIC-value relative to the model with the lowest AIC-value (ΔAIC), its weight, and relative likelihood.

| **Model** | **ΔAIC** | **Akaike Weight** | **Relative LL** |
| --- | --- | --- | --- |
| 7 | 0 | 0.82 | 1 |
| 6 | 3.64 | 0.13 | 0.16 |
| 5 | 5.71 | 0.05 | 0.06 |
| 3 | 13.39 | 0 | 0 |
| 4 | 14.37 | 0 | 0 |
| 2 | 41.64 | 0 | 0 |
| 1 | 102.36 | 0 | 0 |

**Table S2.** Estimated (a) among-individual (**I**) and (b) permanent environmental (**PE**) covariances/correlations (with SE) between two behaviours (aggression and exploration), and IGEs on aggression. The I matrix is derived from the first multivariate model described in the main text; the among-individual covariances are then partitioned in a second model into G matrix (main text, Table 2) and PE matrix presented here. We present covariances (lower-off diagonals) and correlations (upper-off diagonals) for each set of traits. Correlations printed in bold-face are significant (P<0.05) based on likelihood ratio tests derived from the multivariate model detailed in the main text.

| **a. I** | **Aggressiveness** | **Exploration** | **Aggressiveness elicited** |
| --- | --- | --- | --- |
| **Aggressiveness** | - | 0.14 (0.08) | **-0.22** (0.12) |
| **Exploration** | 0.04 (0.02) | - | **0.37** (0.09) |
| **Aggressiveness elicited** | **-0.03** (0.02) | **0.08** (0.02) | - |

| **b. PE** | **Aggressiveness** | **Exploration** | **Aggressiveness elicited** |
| --- | --- | --- | --- |
| **Aggressiveness** | - | 0.34 (0.19) | 0.03 (0.18) |
| **Exploration** | 0.05 (0.03) | - | 0.30 (0.19) |
| **Aggressiveness elicited** | 0.00 (0.02) | 0.04 (0.02) | - |
